# Rethinking Treatment-Resistant Depression: A Systematic Review of Novel Therapeutic Strategies and Precision Medicine Approaches

**DOI:** 10.62641/aep.v53i6.1946

**Published:** 2025-12-17

**Authors:** Safiye Zeynep Tatlı, Murat İlhan Atagün

**Affiliations:** ^1^Department of Psychiatry, Etlik City Hospital, 06170 Ankara, Turkey; ^2^Department of Psychiatry, Faculty of Medicine, Canakkale Onsekiz Mart University, 17020 Canakkale, Turkey

**Keywords:** depressive disorder, treatment-resistant, bipolar disorder, antidepressive agents, ketamine, transcranial magnetic stimulation, precision medicine

## Abstract

**Background:**

Treatment-resistant depression (TRD) is a complex and heterogeneous condition affecting a considerable subset of patients who do not respond to conventional antidepressants. Given the limitations of traditional treatment strategies, there is a growing need for alternative and personalized approaches.

**Objective:**

This review explores the neurobiological underpinnings of TRD and examines the efficacy of emerging pharmacological and neuromodulatory interventions. We also highlight the potential role of the bipolar spectrum in TRD and the need for tailored treatment strategies.

**Methods:**

A systematic review of literature from 2015 to 2025 was conducted using PubMed and Scopus. Studies on TRD treatment modalities, including augmentation strategies, mood stabilizers, atypical antipsychotics, and neuromodulation techniques, were analyzed.

**Results:**

Our findings indicate that novel interventions, such as ketamine, esketamine, psychedelics, and neuromodulation therapies (e.g., repetitive transcranial magnetic stimulation, magnetic seizure therapy) show promise in addressing TRD. Additionally, biomarker-driven and pharmacogenetic approaches may enhance treatment selection and improve outcomes. Evidence suggests that a subset of patients with TRD could fall within the bipolar spectrum, requiring mood stabilizers and antipsychotics rather than standard antidepressant regimens.

**Conclusion:**

A multidisciplinary and precision-based approach is essential for optimizing TRD management. Future research should focus on biomarker-driven treatment selection, artificial intelligence-assisted decision making, and large-scale trials to refine personalized therapeutic strategies.

## Introduction

Treatment-resistant depression (TRD) is a clinical condition characterized by an inadequate response to at least two different classes of antidepressants administered at an optimal dose and duration [1]. A considerable proportion of patients with major depressive disorder (MDD) do not respond to initial antidepressant therapy, and many require multiple treatment attempts before remission is achieved. TRD imposes a substantial burden at both individual and societal levels, leading to a marked decline in quality of life, increased functional impairment and mortality [2]. Currently, three primary pharmacotherapeutic strategies are used for the clinical management of TRD: antidepressant dose optimization, augmentation or combination therapies, and switching pharmacotherapy [3]. However, despite these strategies, a significant number of patients fail to reach full remission, highlighting the need for a better understanding of the biological and clinical mechanisms underlying TRD [3].

The pathophysiology of TRD is complex and multifactorial, extending beyond the classic monoamine deficiency hypothesis to encompass synaptic dysfunction, neuroinflammation, hypothalamic–pituitary–adrenal (HPA) axis dysregulation, and alterations of the glutamatergic system. A growing body of research has explored potential biomarkers to better characterize TRD and guide personalized interventions. These include: (i) genetic polymorphisms (e.g., serotonin transporter gene SLC6A4, brain-derived neurotrophic factor BDNF, FK506-binding protein 5 FKBP5 [4, 5, 6, 7]; (ii) neuroimaging markers (e.g., altered fronto-limbic connectivity, reduced hippocampal volume) [8, 9]; (iii) inflammatory mediators (e.g., interleukin-6, tumor necrosis factor-α, C-reactive protein) [10, 11, 12, 13]; and (iv) neuroendocrine parameters (e.g., cortisol dysregulation) [10, 14]. While none have yet reached routine clinical practice, these biomarkers hold promise for patient stratification and treatment selection in the future.

Large-scale clinical trials highlight the persistence of TRD despite sequential interventions. For instance, The Sequenced Treatment Alternatives to Relieve Depression (STAR*D) study reported that even after four consecutive antidepressant trials, approximately 30% of patients failed to achieve remission [15]. Furthermore, TRD is associated with increased hospitalization rates and higher health-care costs compared to non-resistant depression. In this context, integrating biomarker-informed strategies and personalized medicine approaches may enhance treatment precision and improve patient outcomes [16].

TRD is increasingly recognized as a heterogeneous clinical phenomenon, with some cases potentially representing manifestations within the broader bipolar spectrum. Several studies have identified subthreshold bipolar features in patients diagnosed with TRD, including early onset of depressive symptoms, recurrent episodes, antidepressant-induced mood instability, atypical symptom patterns, and a family history of bipolar disorder [17, 18, 19, 20]. Nuñez* et al*. [21] reported marked clinical and sociodemographic differences between unipolar TRD and bipolar depression, supporting the notion of diagnostic overlap. Moreover, patients with undiagnosed bipolar depression misclassified as TRD may be less responsive to standard antidepressant therapies and may benefit more from mood stabilizers or atypical antipsychotics [20, 22]. Misdiagnosis in such cases can delay appropriate treatment, reduce the likelihood of recovery, and increase the risk of adverse events such as antidepressant-induced mania. Therefore, consideration of bipolar spectrum features in TRD is essential for accurate diagnosis and effective treatment planning. This review incorporates the bipolar spectrum as a conceptual framework to understand the heterogeneity of TRD rather than as a categorical reclassification.

Neurobiological evidence further implicates glutamatergic dysregulation, chronic neuroinflammation, and structural brain changes are key determinants of TRD progression and treatment response [7]. Additionally, clinical factors, such as early-onset depression, frequent mood episodes, comorbid psychiatric disorders, and a history of childhood trauma have been associated with a more persistent and treatment-resistant course of depression [23, 24].

Given the heterogeneity of TRD and its potential—but not yet fully defined—overlap with bipolar spectrum disorders, further research is needed to better understand recovery mechanisms, identify predictive biomarkers, and develop targeted therapeutic strategies. Several novel and emerging interventions are under investigation for their potential to address limitations of conventional antidepressants. These include glutamatergic modulators (e.g., ketamine hydrochloride (Ketalar®, Pfizer Inc., New York, NY, USA) and esketamine nasal spray (Spravato®, Janssen Pharmaceuticals, Titusville, NJ, USA)), psychedelic compounds (e.g., psilocybin (COMPASS Pathways plc, London, UK) and ayahuasca (União do Vegetal, Brasília, Brazil)), anti-inflammatory agents (e.g., celecoxib (Celebrex®, Pfizer Inc., New York, NY, USA) and infliximab (Remicade®, Janssen Biotech Inc., Horsham, PA, USA)), neurostimulation modalities (e.g., repetitive transcranial magnetic stimulation, magnetic seizure therapy), and integrated multimodal approaches. Early evidence suggests that some of these interventions may offer rapid onset of action, novel mechanisms of effect, or sustained benefits in specific TRD subgroups unresponsive to standard treatments.

The present review synthesizes recent findings within this integrative framework, aiming to highlight promising avenues for more personalized and effective treatment strategies in TRD. While the primary focus is on TRD, we also acknowledge the clinical relevance of bipolar spectrum features in certain cases, supported by clinical, neurobiological, and epidemiological evidence indicating considerable diagnostic and therapeutic overlap between these conditions. This perspective has informed our interpretation of subgroup findings likely to fall within the bipolar spectrum and underscores the need for careful diagnostic assessment. Ultimately, further research is required to refine diagnostic boundaries, validate predictive biomarkers, and optimize individualized treatment approaches.

## Methods

This review analyzed TRD by exploring treatment strategies that extend beyond traditional unipolar depression approaches. A systematic literature search of PubMed (National Library of Medicine, Bethesda, MD, USA) and Scopus (Elsevier B.V., Amsterdam, The Netherlands) was performed to identify relevant studies examining TRD, its clinical and neurobiological characteristics, and its treatment strategies within the broader mood disorder spectrum. This systematic review was conducted in accordance with the Preferred Reporting Items for Systematic Reviews and Meta-Analyses (PRISMA) guidelines and was registered in the PROSPERO international register of systematic reviews (ID: CRD420251028607). A structured search was performed in PubMed and Scopus, spanning from 2015 to 2025. The search strategy included the combination of Medical Subject Headings (MeSH) terms and keywords: “treatment-resistant depression”, “bipolar disorder”, “depressive episode”, “mood disorder spectrum”, “treatment response”, “antidepressant resistance”, “augmentation therapy”, “mood stabilizers”, and “atypical antipsychotics”. Boolean operators (AND/OR) were applied to refine the search and retrieve the most relevant studies. Filters were applied to exclude non-English publications, preprints, studies without full-text availability, and non-human studies. A detailed list of search terms and Boolean operators used in this systematic review is provided in **Supplementary Table 1**.

The inclusion criteria were: peer-reviewed studies focusing on TRD; studies investigating alternative treatment strategies, such as mood stabilizers, atypical antipsychotics, and augmentation therapies; studies assessing clinical, neurobiological, or functional recovery outcomes; and systematic reviews, meta-analyses, randomized controlled trials (RCTs), and large observational studies.

The exclusion criteria were: studies exclusively addressing unipolar depression; case reports, opinion pieces, and editorials; studies with inaccessible full text; studies with insufficient details on treatment response and recovery outcomes; studies lacking clear diagnostic criteria for TRD; preprints, non-English studies, and abstract-only publications; pediatric studies and animal research; and studies with unreliable data sharing or methodological quality concerns.

**Fig. 1.**
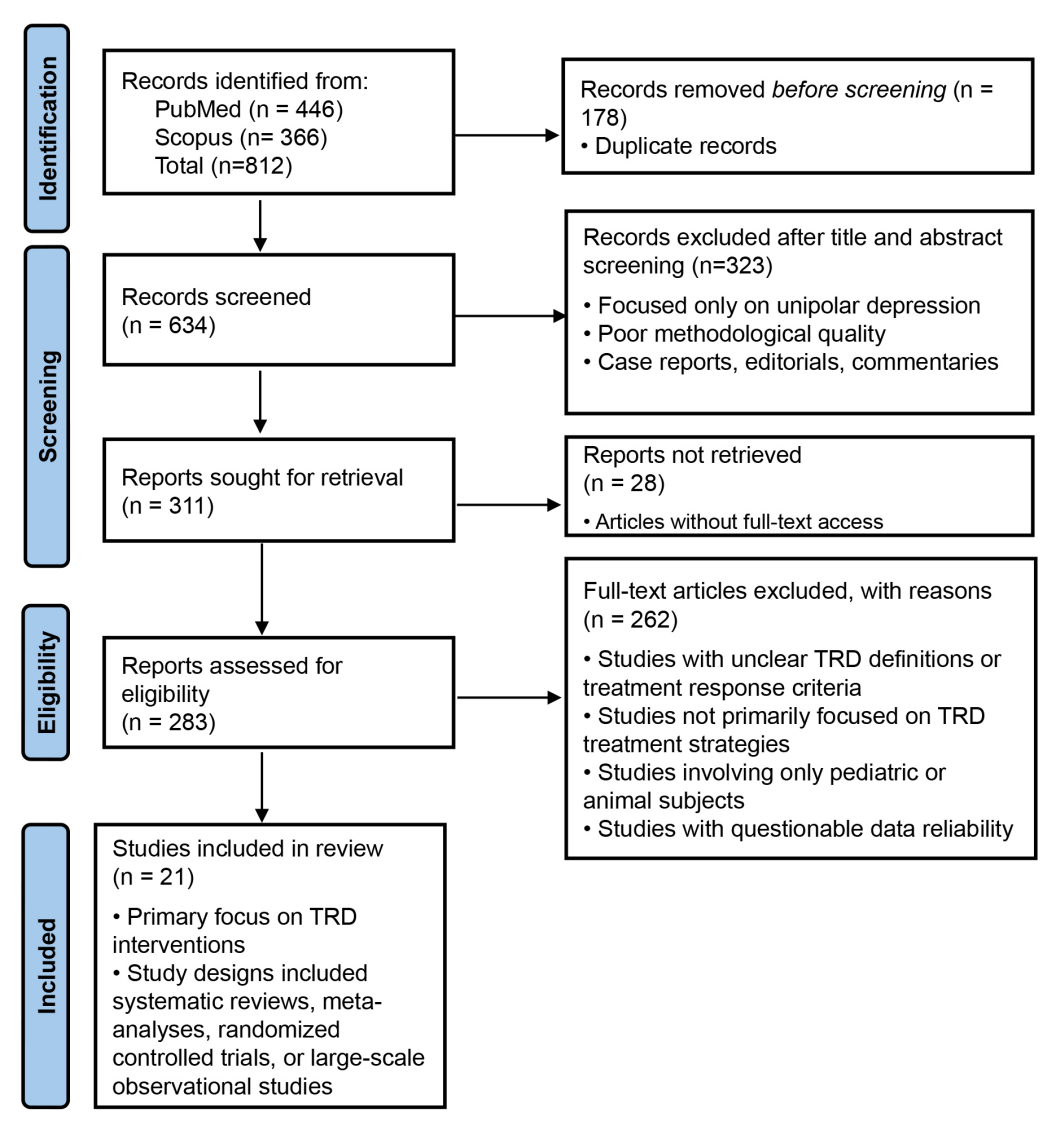
**PRISMA flow diagram illustrating the systematic review process**. PRISMA, Preferred Reporting Items for Systematic Reviews and Meta-Analyses.

A total of 21 studies were identified, which included meta-analyses, systematic reviews, and RCTs. The selection process of studies is shown in the PRISMA flow diagram (Fig. 1) and outlines the number of records identified, screened, assessed for eligibility, and included in the final analysis, with all initial screening steps conducted by a single reviewer. To minimize potential selection bias, predefined eligibility criteria were rigorously applied throughout the process. Additionally, approximately 10% of excluded records (n = 40) were randomly selected and independently reassessed by a second reviewer to ensure consistency and methodological rigor. Data extraction was also conducted by a single reviewer using standardized forms. To enhance data reliability, extracted information from approximately 20% of included studies (n = 5) was randomly selected and independently cross-checked by a second reviewer.

After applying the inclusion and exclusion criteria, 21 studies were deemed suitable for final analysis and are summarized in Table 1 (Ref. [25, 26, 27, 28, 29, 30, 31, 32, 33, 34, 35, 36, 37, 38, 39, 40, 41, 42, 43, 44, 45]). Additional details regarding the pharmacological, psychotherapeutic, and neuromodulatory interventions employed in these studies are provided in **Supplementary Table 2**. Risk of bias was assessed using design-specific tools: Cochrane Risk of Bias 2 (RoB 2.0) tool (version 2.0; Cochrane Collaboration, London, UK) for randomized trials, ROBIS for systematic reviews, and the NIH Quality Assessment tools for observational studies. The results are summarized in **Supplementary Table 3**. Due to the heterogeneity of study designs and methodologies, a narrative synthesis approach was chosen instead of a meta-analysis, which allowed for a structured yet flexible interpretation of findings, focusing on alternative treatment strategies in TRD, TRD within the bipolar spectrum, and predictors of treatment response and functional recovery.

**Table 1.** **Alternative and emerging treatment strategies for TRD**.

Study	Treatment approach	Study type	Findings	Effect size	95% CI	I^2^
Zengin *et al*. (2022) [25]	rTMS	Randomized, Double-Blind, Crossover Study	rTMS was effective and well-tolerated in TRBD, with significant reductions in HAM-D and BDI scores.	0.67–0.68 (Cohen’s d)	NR	NA
Scott *et al*. (2023) [26]	Augmentation & Combination Therapy	Systematic Review & Meta-Analysis	Augmentation strategies (e.g., CBT, ketamine, risperidone) showed highest effect sizes; evidence remains inconsistent across treatments.	CBT: 1.58 (SMD)	1.09–2.07	89%
				Ketamine: 1.48 (SMD)	1.23–1.73	74%
				Risperidone: 1.42 (SMD)	1.29–1.61	72%
Palhano-FOntes *et al*. (2019) [27]	Ayahuasca (Psychedelic)	Randomized, Placebo-Controlled Trial	Significant antidepressant effects of ayahuasca observed, with rapid onset and sustained response at day 7.	0.98 (SMD)	0.21–1.75	NA
Zakhour *et al*. (2020) [28]	CBT	Systematic Review	CBT combined with pharmacotherapy reduced depressive symptoms; effect maintained for up to 12 months.	NA	NR	NA
Fedgchin *et al*. (2019) [29]	Esketamine Nasal Spray	Randomized, Double-Blind, Active-Controlled Study	Esketamine nasal spray showed rapid but inconsistent efficacy; safety concerns included nausea and dissociation.	56 mg: –4. (Diff. of LS means)	–7.67 to –0.49	NA
				84 mg: –3.2 (Diff. of LS means)	–6.88 – 0.45	
Cladder-Micus *et al*. (2018) [30]	MBCT	Randomized Controlled Trial	MBCT showed higher remission rates, improvements in mindfulness skills and self-compassion, but non-significant reductions in depressive symptoms.	0.35–0.45 (SMD)	NR	NA
Ijaz *et al*. (2018) [31]	Psychological Therapies (CBT, IPT, DBT)	Systematic Review (Cochrane Review)	CBT and other psychological therapies improved remission rates and reduced depressive symptoms when added to usual care, but long-term benefits were less clear.	–0.40 (SMD)	–0.65 to –0.14	37%
Lenze *et al*. (2023) [32]	Antidepressant Augmentation vs Switching	Randomized, Open-Label Trial	Augmentation with aripiprazole was more effective than switching to bupropion in improving well-being in geriatric TRD; lithium and nortriptyline had similar effects.	0.37 (SMD)	0.07–0.67	NA
Daly *et al*. (2019) [33]	Esketamine Nasal Spray	Phase 3 Randomized, Double-Blind Study	Esketamine nasal spray significantly delayed relapse compared to placebo, reducing relapse risk by 51–70%.	0.49 (HR)	0.29–0.84	NA
Nuñez *et al*. (2022) [34]	Augmentation Strategies	Systematic Review & Network Meta-Analysis	Atypical antipsychotics, thyroid hormones, and dopamine-related agents were the most effective augmentation strategies; acceptability was lower with certain agents (e.g., ziprasidone, mirtazapine).	Response: 1.18–1.90 (RR, range across agents)	1.03–3.11	9.8%
				Remission: 1.44–1.91 (RR, range across agents)	1.00–3.52	2.4%
Phillips *et al*. (2020) [35]	Single & Repeated Ketamine Infusions	Randomized, Double-Blind, Crossover Study	Single ketamine infusion significantly reduced suicidal ideation, effects sustained for 7 days; repeated infusions led to cumulative suicidal ideation reduction.	0.83 (SMD)	NR	NA
Mcmullen *et al*. (2021) [36]	Prolonging Ketamine’s Efficacy	Systematic Review & Meta-Analysis	Repeated-dose IV ketamine showed prolonged antidepressant effects, but no other modality effectively extended its efficacy.	NA	NR	NA
Papakostas *et al*. (2024) [37]	Aripiprazole, rTMS vs. Venlafaxine XR	RCT	rTMS augmentation was superior to switching antidepressants for TRD, while aripiprazole showed mixed results.	rTMS: 4.17 (MD)	NR	NA
				Aripiprazole: 1.72 (MD)	NR	NA
Rost *et al*. (2024) [38]	Data-Driven TRD Approach	Observational, Data-Driven Study	TRD is a complex disorder with high psychiatric comorbidities (82.9%) and significant physical health problems (69.8%).	NA	NR	NA
Ledesma-Corvi *et al*. (2024) [39]	Rapid Treatment for Adolescents	Review and Experimental Studies	Ketamine, psychedelics, and cannabinoids may provide rapid relief for adolescent TRD; clinical validation needed.	NA	NR	NA
Strawn *et al*. (2020) [40]	TRD in Adolescents	Observational Study with ATR	Adolescents with TRD had a median CGI-S score of 5, high comorbidities, and prolonged illness duration; ATR stratified resistance levels.	NA	NR	NA
Daly *et al*. (2018) [41]	Intranasal Esketamine	Phase 2 Trial	Rapid-onset antidepressant effects; significant MADRS score reduction; dose-dependent efficacy observed.	28 mg: –4.2 (MD)	–7.67 to –0.79	NA
				56 mg: –6.3 (MD)	–9.71 to –2.88	
				84 mg: –9.0 (MD)	–12.53 to –5.52	
Jiang *et al*. (2021) [42]	MST	Cochrane Review	MST is a potential alternative to ECT with fewer cognitive side effects; limited high-quality evidence.	0.71 (MD)	–2.23–3.65	7%
Glue *et al*. (2024) [43]	Extended-Release Ketamine	Extended-Release Ketamine Trial	Extended-release ketamine had sustained efficacy with lower relapse rates; well-tolerated.	–6.1 (MD)	1.00–11.16	NA
Jha *et al*. (2024) [44]	Ketamine vs. ECT	ELEKT-D Trial	Ketamine demonstrated non-inferiority to ECT for nonpsychotic TRD; outpatients showed greater improvement with ketamine.	NA	NR	NA
Oliveira-Maia *et al*. (2024) [45]	Real-World Outcomes in TRD	Systematic Real-World Review	Real-world studies show heterogeneous outcomes; lack of standardized assessment measures in TRD practice.	NA	NR	NA

CI, Confidence interval; NA, Not applicable; NR, Not reported; SMD, Standardized mean difference; MD, Mean difference; HR, Hazard ratio; rTMS, repetitive transcranial magnetic stimulation; TRBD, treatment-resistant bipolar depression; HAM-D, Hamilton Depression Rating Scale; CBT, cognitive-behavioral therapy; MBCT, mindfulness-based cognitive therapy; IPT, interpersonal psychotherapy; DBT, dialectical behavior therapy; ATR, Antidepressant Treatment Record; MADRS, Montgomery–Åsberg Depression Rating Scale; MST, magnetic seizure therapy; ECT, electroconvulsive therapy.

To ensure the integrity of this review and to avoid duplication, primary studies included in selected review articles were carefully examined. Of the 21 studies, eight were review articles. Only two studies (Daly* et al*. [41] and Fedgchin* et al*. [29]) overlapped with a review by Scott* et al*. [26], which included 111 studies; however, both were independently identified from our search and met the inclusion criteria. There were no other duplications and since this overlap constitutes only a small portion of the overall dataset, it is unlikely to introduce bias or affect the validity of our findings.

To assess the effect of various treatment strategies on TRD, effect sizes (including standardized mean difference [SMD], odds ratio [OR], and hazard ratio [HR]), 95% confidence intervals (CI), and heterogeneity measures (I^2^) were extracted where reported. Due to the heterogeneity of study designs and outcomes, a meta-analysis was not feasible, therefore, a narrative synthesis was conducted.

## Results

TRD poses a major challenge in clinical practice due to its heterogeneous etiology and the poor response of many patients to conventional antidepressants. Given its complexity, a wide range of strategies have been investigated, including pharmacological augmentation, neuromodulatory techniques, psychotherapeutic approaches, as well as novel and emerging agents. Conventional strategies such as dose optimization or switching antidepressants often prove insufficient in severe or chronic cases, necessitating multimodal or targeted interventions. Table 1 summarizes the effect sizes, confidence intervals, and heterogeneity measures for the emerging and recently studied interventions identified in our systematic search. Details are provided in **Supplementary Table 3**. Most studies showed low risk of bias; however, “some concerns” were noted in a subset of RCTs, primarily due to limitations in blinding or reporting.

### Pharmacological Interventions

Ketamine hydrochloride (Ketalar®, Pfizer Inc., New York, NY, USA) and esketamine nasal spray (Spravato®, Janssen Pharmaceuticals, Titusville, NJ, USA) demonstrated the most robust short-term efficacy among pharmacological options, with rapid onset of action. These agents represent an alternative treatment pathway for patients with TRD who remain unresponsive to conventional pharmacotherapy, including those who have failed multiple prior antidepressant trials. Augmentation strategies, particularly with atypical antipsychotics, also showed beneficial effects on treatment response. Novel agents, particularly psilocybin-a classic psychedelic, show potential as an emerging treatment option for TRD; however, current evidence is limited, and further studies are required to establish its long-term safety and efficacy.

### Neuromodulatory Interventions

Neuromodulatory strategies, including repetitive transcranial magnetic stimulation (rTMS), and magnetic seizure therapy (MST) have demonstrated efficacy in the treatment of TRD. MST has emerged as a potential alternative to ECT, offering comparable antidepressant effects with fewer cognitive risks in preliminary studies. rTMS demonstrated sustained antidepressant effects, particularly in chronic TRD. Maintenance protocols and combination with pharmacotherapy appeared to prolong benefits.

### Psychotherapeutic Interventions

Psychotherapeutic approaches, including cognitive behavioral therapy (CBT) and mindfulness-based cognitive therapy (MBCT), showed small-to-moderate benefits when delivered adjunctive to usual care in TRD. Evidence suggests CBT provides the strongest support for sustained medium- to long-term benefits, while MBCT may improve metacognitive and emotional regulation capacities.

### Considerations for Bipolar-Spectrum TRD

A subset of patients with TRD in included studies exhibited clinical features suggestive of bipolar-spectrum illness, as reflected by treatment response patterns. In such cases, mood stabilizers and atypical antipsychotics were preferred over antidepressant monotherapy to reduce the risk of mood switching.

Table 1 presents the effect sizes and statistical details for the included studies, while Table 2 (Ref. [21, 25, 27, 28, 29, 30, 31, 32, 33, 35, 36, 37, 41, 42, 43, 44, 45]) provides a structured grouping of these interventions, enabling comparison across pharmacological, neuromodulatory, and psychotherapeutic modalities.

**Table 2.** **Overview of evidence-based and novel approaches in TRD treatment**.

Treatment strategy	Mechanism	Findings	Key studies
Ketamine & Esketamine [29, 33, 35, 36, 41, 43, 44]	NMDA antagonism	Ketamine and esketamine demonstrate rapid antidepressant effects with significant MADRS reduction; ketamine showed non-inferiority to ECT in some trials; esketamine is FDA-approved for TRD but requires careful monitoring for dissociation and abuse potential.	RCT on extended-release ketamine
			Intranasal esketamine trials
			Single & repeated ketamine infusions
			Ketamine vs. ECT
			Systematic review on ketamine efficacy
MST [42]	Magnetic seizure induction	MST offers a potential alternative to ECT with fewer cognitive impairments; efficacy needs further validation.	Cochrane Review on comparative trials with ECT
rTMS [25, 37]	Cortical stimulation	rTMS demonstrated sustained antidepressant effects in TRD patients; effectiveness varied based on stimulation parameters.	rTMS comparative trials
			rTMS efficacy in TRBD
Psychedelics [27]	Serotonin receptor modulation	Ayahuasca showed rapid antidepressant effects; long-term efficacy still requires validation.	Ayahuasca placebo-controlled trial
Addition of psychotherapy to treatment as usual [28, 30, 31]		CBT, IPT, and DBT, when combined with usual treatment, improved remission rates and reduced depressive symptoms; long-term benefits remain unclear. MBCT has also demonstrated effectiveness in reducing relapse rates and improving emotional regulation.	Cochrane Review on psychological therapies
			Systematic review on CBT for TRD
			MBCT trial for chronic TRD
Real-World Treatment Outcomes [45]		Real-world studies show heterogeneous TRD treatment outcomes; patient-centered metrics are underdeveloped.	Systematic real-world TRD study
Bipolar Spectrum Considerations in TRD [21, 32]		A subset of TRD patients may belong to the bipolar spectrum; mood stabilizers and atypical antipsychotics may be preferable over antidepressants.	Emerging evidence in mood disorder classification

NMDA, N-methyl-D-aspartate; MADRS, Montgomery–Åsberg Depression Rating Scale; ECT, electroconvulsive therapy; RCT, randomized controlled trial; MST, magnetic seizure therapy; rTMS, repetitive transcranial magnetic stimulation; CBT, cognitive-behavioral therapy; IPT, interpersonal psychotherapy; DBT, dialectical behavior therapy; MBCT, mindfulness-based cognitive therapy; TRD, Treatment-resistant depression.

## Discussion

TRD is increasingly recognized as a clinically and biologically heterogeneous condition comprising distinct subgroups with varying treatment needs [38, 45]. While conventionally defined as the non-response to at least two adequate trials of antidepressants, resistance mechanisms are diverse and involve neuroinflammation, HPA axis dysregulation, glutamatergic abnormalities, genetic and epigenetic factors, and structural and functional brain alterations. Neuroimaging studies have particularly implicated the dorsolateral prefrontal cortex, anterior cingulate cortex, and hippocampus in TRD pathophysiology [10, 46, 47].

This heterogeneity has led to a growing emphasis on mechanism-based treatment strategies. The studies presented in Tables 1,2 show that conventional pharmacological approaches often remain insufficient for many patients with TRD, highlighting the increasing relevance of alternative and biologically-informed interventions. In recent years, novel treatment options—including glutamatergic agents, psychedelics, and neuromodulation therapies—have gained attention for their rapid and unique mechanisms of action.

Among these, N-methyl-D-aspartate (NMDA)-modulating agents such as ketamine and esketamine have shown rapid antidepressant effects, particularly in bipolar-spectrum TRD, where glutamatergic dysfunction is implicated. Neuroimaging and spectroscopy studies in bipolar depression report altered glutamate and N-acetylaspartate levels in prefrontal and anterior cingulate cortices, indicating excitotoxic stress and impaired neuroenergetics [48]. Additionally, functional connectivity data suggest that ketamine responders exhibit rapid normalization of disrupted networks such as the default mode and salience networks following NMDA modulation [49], while trajectory models suggest a progression from early glutamate excess to NMDA hypofunction in bipolar disorder, which helps explain the stabilizing effects of NMDA antagonists [50]. Real-world data from the multicentric REAL-ESK study further support this view, showing that esketamine yields comparable antidepressant effects in bipolar and unipolar TRD, without an increased risk of manic switch [51]. While these agents show therapeutic promise, their dissociative side effects and abuse potential necessitate careful monitoring in clinical use [29, 33, 41].

In parallel, serotonergic psychedelics such as ayahuasca have emerged as alternative glutamatergic modulators. Acting primarily via 5-hydroxytryptamine 2A receptor (5-HT2A) receptor agonism, they are proposed to indirectly facilitate glutamatergic activity via thalamocortical and cortical pyramidal pathways, facilitating neural circuit reorganization and enhance synaptic plasticity, although this mechanism remains under active investigation. RCTs have demonstrated rapid and sustained antidepressant effects, positioning them as promising candidates for TRD. However, concerns regarding long-term safety currently restrict their use to controlled clinical environments [27]. In structured therapeutic settings, recent trials suggest an overall acceptable short-term safety profile. For example, Palhano-Fontes* et al*. (2019) [27] reported nausea and vomiting in 57% of ayahuasca-treated patients, along with transient dizziness, paresthesia, and thermoregulatory changes, without serious or persistent psychiatric complications. Carhart-Harris* et al*. (2012) [52], in a small open-label study of patients with TRD, found psilocybin to be generally well tolerated. Reported adverse reactions were transient anxiety during drug onset (all patients), transient confusion or thought disorder, mild and transient nausea, and transient headache. A systematic review by Breeksema* et al*. (2022) [53], encompassing 44 clinical studies involving serotonergic psychedelics (including psilocybin and ayahuasca), confirmed that most adverse events were mild, self-limiting, and did not require medical intervention. Importantly, no cases of persistent psychosis, mania, or hallucinogen persisting perception disorder were reported. Nonetheless, the review emphasized that long-term safety remains insufficiently characterized due to limited follow-up and lack of standardized adverse event monitoring [53]. These findings underscore the need for improved and systematic adverse event tracking in future studies, particularly when applied to vulnerable populations such as individuals with TRD.

Neuromodulation strategies provide additional options for TRD, particularly in pharmacotherapy-refractory cases. ECT remains the most effective intervention for severe TRD, and is especially beneficial in bipolar depression, but its use is limited by cognitive adverse effects [54]. MST offers similar efficacy with fewer cognitive side effects [42]. rTMS also shows sustained efficacy, particularly when individualized parameters are applied [25, 37]. Both rTMS and MST exert their effects by modulating cortical excitability through repeated electromagnetic pulses, typically targeting the dorsolateral prefrontal cortex. These interventions promote gradual reorganization of dysfunctional fronto-limbic circuits via long-term potentiation-like (LTP) mechanisms, resulting in slower but potentially more durable improvements [25, 42, 55]. Vagus nerve stimulation and deep brain stimulation (including closed-loop neuromodulation strategies) are emerging as promising neuromodulation strategies for advanced-stage TRD [56, 57, 58]; however, no studies involving these methods met our inclusion criteria.

Given the diversity of emerging interventions, it is essential to understand their underlying neurobiological mechanisms to guide more effective and individualized treatment selection. Synthesizing these mechanistic pathways not only clarifies their therapeutic rationale but also supports a precision-guided approach to care. NMDA-modulating agents rapidly enhance synaptic plasticity via glutamate surge, AMPA receptor activation, and downstream mTOR/BDNF signaling [59]. Psychedelics exert similar effects via indirect modulation of glutamatergic pathways and disruption of the DMN, promoting cognitive flexibility and emotional relearning [52]. In contrast, neuromodulation techniques gradually reorganize fronto-limbic circuits through long-term potentiation-like mechanisms [55, 60]. Although direct comparisons between treatment modalities were not within the scope of our review, these neurobiological and temporal differences may help contextualize the variability in clinical responses reported across studies. These varied approaches, while neurobiologically distinct, converge on restoring plasticity and network integration—core targets in TRD—and underscore the need for biologically stratified treatment algorithms.

Psychotherapeutic interventions demonstrate moderate efficacy in TRD, particularly when combined with pharmacotherapy. In the Cochrane review by Ijaz *et al*. (2018) [31], six randomized controlled trials showed that psychotherapy added to treatment-as-usual significantly improved self-reported depressive symptoms (SMD = –0.40; 95% CI: –0.65 to –0.14). Adjunctive psychotherapy also improved short-term response (RR = 1.80; 95% CI: 1.20–2.69) and remission (RR = 1.92; 95% CI: 1.46–2.52) rates, with dropout rates comparable to controls. Although trial sizes were modest and at some risk of detection bias, evidence from follow-up assessments suggests sustained benefits over medium (12 months) and long-term (up to 46 months) periods, especially with cognitive-behavioral therapy (CBT) [31]. Mindfulness-Based Cognitive Therapy (MBCT) has also shown promise as an adjunctive treatment in chronic and TRD. In a multicenter RCT, Cladder-Micus* et al*. (2018) [30]reported that MBCT added to treatment-as-usual significantly increased remission rates (χ^2^(2) = 4.25, φ = 0.22, *p* = 0.04) and led to improvements in rumination (*d* = 0.39), quality of life (*d* = 0.42), mindfulness skills (*d* = 0.73), and self-compassion (*d* = 0.64). While intent-to-treat analyses did not show significant symptom reductions in depressive symptom severity, per-protocol analyses revealed a significant effect (*d* = 0.45). These results suggest that MBCT’s principal benefits may be mediated through enhancements in metacognitive abilities and emotional regulation, with indirect effects on depressive symptoms. Follow-up data up to six months indicate potential maintenance of these gains [30]. In a broader synthesis, Zakhour* et al*. (2020) [28] reviewed eight studies (four adult RCTs, two adolescent RCTs, one open trial, and one case report) and concluded that CBT combined with pharmacotherapy consistently reduced depressive symptoms in TRD, particularly in cases of partial antidepressant response. The review highlighted the influence of prior treatment history, chronicity of resistance, and comorbidities such as anxiety or personality disorders on therapeutic outcomes, though heterogeneity precluded pooled effect estimates [28]. Overall, while psychotherapies offer meaningful adjunctive benefits in TRD, their standalone efficacy remains limited. The cumulative evidence favors an integrated, multimodal approach, combining pharmacological, psychotherapeutic, and neuromodulatory interventions. Moving forward, tailoring psychotherapeutic interventions to individual clinical characteristics, illness course, and treatment history, potentially in combination with pharmacological and neuromodulatory strategies, may enhance both symptom remission and long-term functional recovery.

Despite advancements in biological and psychological treatments, real-world outcomes remain highly variable. While some patients with TRD show long-term improvement, many continue to experience relapsing or chronic episodes, with only partial or temporary response to treatment [45]. This variability underscores the need for personalized, long-term treatment strategies that go beyond acute symptom control. To guide clinical decision making, future models of care should incorporate longitudinal monitoring, functional recovery metrics, and patient-centred outcome measures. Integration of clinical, biological, and psychosocial data is key to achieving sustained remission and improved quality of life.

Among the heterogeneous TRD population, a particularly important subgroup includes patients with features of the bipolar spectrum. A growing body of evidence suggests that a subset of patients diagnosed with TRD exhibit subthreshold or misdiagnosed bipolar traits, including early-onset depression, frequent mood episodes, antidepressant-induced instability, or a family history of bipolar disorder. In these cases, conventional antidepressants may not only be ineffective, but could potentially be harmful, leading to treatment-emergent mania, mood destabilization, or rapid cycling. For such individuals, mood stabilizers and atypical antipsychotics are generally preferred instead of antidepressant monotherapy [20, 32, 34].

Accurate diagnosis relies on careful longitudinal assessment of clinical history, family psychiatric history, and screening for mixed or hypomanic features. However, despite increasing awareness of this overlap, routine screening for bipolarity in patients with TRD remains inconsistent. Structured interviews, bipolar spectrum questionnaires, and ongoing mood tracking should be incorporated into clinical practice to reduce misclassification and improve treatment alignment. Reframing a portion of TRD within the bipolar spectrum has major clinical implications. Recognizing this overlap facilitates tailored interventions, reduces the risk of inappropriate treatment, and promotes more durable outcomes. This perspective is increasingly acknowledged in recent clinical guidelines.

International clinical guidelines emphasize the importance of personalized, stepwise approaches in TRD management. The Canadian Network for Mood and Anxiety Treatments (CANMAT 2023) and the UK National Institute for Health and Care Excellence (NICE 2022) provide evidence-based recommendations. CANMAT supports augmentation strategies (e.g., aripiprazole, brexipiprazole, lithium, and T3) for partial responders, while NICE emphasizes systematic evaluation of resistance and caution in antidepressant use among bipolar-spectrum cases. Both guidelines recommend mood stabilizers (e.g., lithium and lamotrigine) or second-generation antipsychotics for patients with inadequate response to antidepressants [1, 61].

Based on the findings of this review, we emphasize that patients with TRD should be systematically evaluated for bipolar-spectrum features, and that treatment planning should prioritize mood stabilizers and antipsychotics instead of antidepressant monotherapy when clinically indicated. Nonetheless, it is important to interpret these findings in light of certain methodological limitations. The selected studies varied in design, patient populations, diagnostic criteria, and treatment approaches, making direct comparisons difficult. Many studies focused on short-term outcomes rather than long-term remission, limiting conclusions about sustained effects. Additionally, inconsistent definitions of TRD across studies and heterogeneous methodologies reduced comparability and hindered synthesis. High heterogeneity (I^2^>40%) observed in some studies, along with inconsistent reporting of effect sizes and confidence intervals, complicates the interpretation of aggregated findings. Furthermore, the exclusion of non-English and unpublished literature may have introduced selection bias. Our search strategy, limited to PubMed and Scopus, and single-reviewer screening also represent methodological constraints. Emerging treatment modalities—e.g., psychedelics and pharmacogenetic-guided therapy—are still supported by limited evidence and require further validation. Some of the included studies also suffered from small sample sizes or had industry funding, which may introduce bias. One included study has post-publication comments on PubPeer; its inclusion was maintained but interpreted cautiously. Despite these limitations, this review provides a comprehensive and up-to-date synthesis of current and emerging therapeutic strategies for TRD, with particular attention to its potential overlap with the bipolar spectrum.

A comprehensive treatment strategy for TRD should address biological, psychological, and environmental dimensions. In addition to pharmacotherapy and neuromodulation, emerging strategies, such as psychedelics and biomarker-based interventions, represent promising avenues. For optimal clinical outcomes, there is a clear need to expand the implementation of personalized treatment protocols and to conduct long-term efficacy studies. Future research should focus on better characterization of TRD subtypes, refinement of neurobiological biomarkers, and evaluation of novel pharmacological agents. Large-scale RCTs are also necessary to establish the effectiveness of combination and sequential treatment approaches.

Building on this perspective, recent innovations are enabling more targeted and better personalized treatment approaches for TRD. Pharmacogenetic testing (e.g., cytochrome P450 (CYP450), BDNF, and FKBP5 polymorphisms) [62, 63, 64, 65], neurobiological biomarkers (e.g., C-reactive protein (CRP), interleukin-6 (IL-6), and and tumor necrosis factor-alpha (TNF-α)) [47], and neuroimaging tools (e.g., functional magnetic resonance imaging (fMRI) and electroencephalography (EEG)) [66] are promising for identifying TRD subtypes and predicting treatment response. Future treatments could involve NMDA antagonists, AMPA receptor modulators, opioid receptor modulators, anti-inflammatory agents, and psychedelics, which diverge from traditional monoaminergic mechanisms [16, 67]. In parallel, artificial intelligence-based decision-support systems are expected to guide individualized treatment selection more efficiently [68]. In particular, accurate diagnosis and stratification of patient subgroups is essential for the effective implementation of these technologies. Early identification of bipolar spectrum features in patients with TRD is critical for guiding appropriate treatment decisions. Emerging advances in pharmacogenetics and neuroimaging hold promise for reducing misdiagnosis and supporting more precise, individualized interventions.

## Conclusion

This systematic review highlights that glutamatergic agents—particularly ketamine and esketamine—are among the most promising interventions for TRD, with emerging evidence of rapid antidepressant effects, especially in individuals with bipolar-spectrum features. Neurostimulation methods and multimodal strategies also show potential, although the strength of evidence varies across subgroups. These findings emphasize the importance of personalized treatment planning based on diagnostic profile, stage of resistance, and comorbidities.

Future research should focus on refining TRD subtypes, validating neurobiological and pharmacogenetic biomarkers, and testing precision medicine models through large-scale clinical trials. The integration of artificial intelligence and biomarker-informed care has the potential to enhance treatment matching and long-term outcomes. Ultimately, a stratified and data-driven framework will be key to improving the quality of life in individuals with TRD.

This systematic review was registered in the PROSPERO database (CRD420251028607). No amendments were made to the study methodology following registration. No formal protocol was published. The template forms, extracted data, and analytic materials used in this review are not publicly available. This review did not receive any external funding. The authors declare no conflicts of interest relevant to this review.

## Supplementary Material

Supplementary material associated with this article can be found, in the online version, at https://doi.org/10.62641/aep.v53i6.1946.

## References

[1] Lam RW, Kennedy SH, Adams C, Bahji A, Beaulieu S, Bhat V (2024). Canadian Network for Mood and Anxiety Treatments (CANMAT) 2023 Update on Clinical Guidelines for Management of Major Depressive Disorder in Adults: Réseau canadien pour les traitements de l’humeur et de l’anxiété (CANMAT) 2023: Mise à jour des lignes directrices cliniques pour la prise en charge du trouble dépressif majeur chez les adultes. *Canadian Journal of Psychiatry. Revue Canadienne De Psychiatrie*.

[2] DiBernardo A, Lin X, Zhang Q, Xiang J, Lu L, Jamieson C (2018). Humanistic outcomes in treatment resistant depression: a secondary analysis of the STAR*D study. *BMC Psychiatry*.

[3] Souery D, Papakostas GI, Trivedi MH (2006). Treatment-resistant depression. *The Journal of Clinical Psychiatry*.

[4] Bonvicini C, Minelli A, Scassellati C, Bortolomasi M, Segala M, Sartori R (2010). Serotonin transporter gene polymorphisms and treatment-resistant depression. *Progress in Neuro-psychopharmacology & Biological Psychiatry*.

[5] Anttila S, Huuhka K, Huuhka M, Rontu R, Hurme M, Leinonen E (2007). Interaction between 5-HT1A and BDNF genotypes increases the risk of treatment-resistant depression. *Journal of Neural Transmission (Vienna, Austria: 1996)*.

[6] Zhang Y, Yue W, Li J (2024). The association of FKBP5 gene polymorphism with genetic susceptibility to depression and response to antidepressant treatment- a systematic review. *BMC Psychiatry*.

[7] Na K-S, Choi K-Y, Kim Y-K, Kim Y-K (2017). Treatment-resistant depression: neurobiological etiology and pharmacological treatment strategies. *Major Depressive Disorder: Risk Factors, Characteristics and Treatment Options*.

[8] Miola A, Meda N, Perini G, Sambataro F (2023). Structural and functional features of treatment-resistant depression: A systematic review and exploratory coordinate-based meta-analysis of neuroimaging studies. *Psychiatry and Clinical Neurosciences*.

[9] Maller JJ, Daskalakis ZJ, Thomson RHS, Daigle M, Barr MS, Fitzgerald PB (2012). Hippocampal volumetrics in treatment-resistant depression and schizophrenia: the devil’s in de-tail. *Hippocampus*.

[10] Mancuso E, Sampogna G, Boiano A, Della Rocca B, Di Vincenzo M, Lapadula MV (2023). Biological correlates of treatment resistant depression: a review of peripheral biomarkers. *Frontiers in Psychiatry*.

[11] Raison CL, Rutherford RE, Woolwine BJ, Shuo C, Schettler P, Drake DF (2013). A randomized controlled trial of the tumor necrosis factor antagonist infliximab for treatment-resistant depression: the role of baseline inflammatory biomarkers. *JAMA Psychiatry*.

[12] Strawbridge R, Arnone D, Danese A, Papadopoulos A, Herane Vives A, Cleare AJ (2015). Inflammation and clinical response to treatment in depression: A meta-analysis. *European Neuropsychopharmacology: the Journal of the European College of Neuropsychopharmacology*.

[13] Chamberlain SR, Cavanagh J, de Boer P, Mondelli V, Jones DNC, Drevets WC (2019). Treatment-resistant depression and peripheral C-reactive protein. *The British Journal of Psychiatry: the Journal of Mental Science*.

[14] Wu X, Dai B, Yan F, Chen Y, Xu Y, Xia Q (2022). Serum Cortisol, Nesfatin-1, and IL-1β: Potential Diagnostic Biomarkers in Elderly Patients with Treatment-Resistant Depression. *Clinical Interventions in Aging*.

[15] Rush AJ, Trivedi MH, Wisniewski SR, Nierenberg AA, Stewart JW, Warden D (2006). Acute and longer-term outcomes in depressed outpatients requiring one or several treatment steps: a STAR*D report. *The American Journal of Psychiatry*.

[16] Kim Y-K (2019). *Treatment Resistance in Psychiatry: Risk Factors, Biology, and Management*.

[17] Perugi G, Pacchiarotti I, Mainardi C, Verdolini N, Menculini G, Barbuti M (2019). Patterns of response to antidepressants in major depressive disorder: Drug resistance or worsening of depression are associated with a bipolar diathesis. *European Neuropsychopharmacology: the Journal of the European College of Neuropsychopharmacology*.

[18] Angst J, Azorin JM, Bowden CL, Perugi G, Vieta E, Gamma A (2011). Prevalence and characteristics of undiagnosed bipolar disorders in patients with a major depressive episode: the BRIDGE study. *Archives of General Psychiatry*.

[19] Mitchell PB, Wilhelm K, Parker G, Austin MP, Rutgers P, Malhi GS (2001). The clinical features of bipolar depression: a comparison with matched major depressive disorder patients. *The Journal of Clinical Psychiatry*.

[20] Fogelson DL, Kagan BL (2022). Bipolar spectrum disorder masquerading as treatment resistant unipolar depression. *CNS Spectrums*.

[21] Nuñez NA, Comai S, Dumitrescu E, Ghabrash MF, Tabaka J, Saint-Laurent M (2018). Psychopathological and sociodemographic features in treatment-resistant unipolar depression versus bipolar depression: a comparative study. *BMC Psychiatry*.

[22] Vieta E, Colom F (2011). Therapeutic options in treatment-resistant depression. *Annals of Medicine*.

[23] Buoli M, Capuzzi E, Caldiroli A, Ceresa A, Esposito CM, Posio C (2022). Clinical and Biological Factors Are Associated with Treatment-Resistant Depression. *Behavioral Sciences (Basel, Switzerland)*.

[24] McIntyre RS, Alsuwaidan M, Baune BT, Berk M, Demyttenaere K, Goldberg JF (2023). Treatment-resistant depression: definition, prevalence, detection, management, and investigational interventions. *World Psychiatry: Official Journal of the World Psychiatric Association (WPA)*.

[25] Zengin G, Topak OZ, Atesci O, Culha Atesci F (2022). The Efficacy and Safety of Transcranial Magnetic Stimulation in Treatment-Resistant Bipolar Depression. *Psychiatria Danubina*.

[26] Scott F, Hampsey E, Gnanapragasam S, Carter B, Marwood L, Taylor RW (2023). Systematic review and meta-analysis of augmentation and combination treatments for early-stage treatment-resistant depression. *Journal of Psychopharmacology (Oxford, England)*.

[27] Palhano-Fontes F, Barreto D, Onias H, Andrade KC, Novaes MM, Pessoa JA (2019). Rapid antidepressant effects of the psychedelic ayahuasca in treatment-resistant depression: a randomized placebo-controlled trial. *Psychological Medicine*.

[28] Zakhour S, Nardi AE, Levitan M, Appolinario JC (2020). Cognitive-behavioral therapy for treatment-resistant depression in adults and adolescents: a systematic review. *Trends in Psychiatry and Psychotherapy*.

[29] Fedgchin M, Trivedi M, Daly EJ, Melkote R, Lane R, Lim P (2019). Efficacy and Safety of Fixed-Dose Esketamine Nasal Spray Combined With a New Oral Antidepressant in Treatment-Resistant Depression: Results of a Randomized, Double-Blind, Active-Controlled Study (TRANSFORM-1). *The International Journal of Neuropsychopharmacology*.

[30] Cladder-Micus MB, Speckens AEM, Vrijsen JN, T Donders AR, Becker ES, Spijker J (2018). Mindfulness-based cognitive therapy for patients with chronic, treatment-resistant depression: A pragmatic randomized controlled trial. *Depression and Anxiety*.

[31] Ijaz S, Davies P, Williams CJ, Kessler D, Lewis G, Wiles N (2018). Psychological therapies for treatment-resistant depression in adults. *The Cochrane Database of Systematic Reviews*.

[32] Lenze EJ, Mulsant BH, Roose SP, Lavretsky H, Reynolds CF, Blumberger DM (2023). Antidepressant Augmentation versus Switch in Treatment-Resistant Geriatric Depression. *The New England Journal of Medicine*.

[33] Daly EJ, Trivedi MH, Janik A, Li H, Zhang Y, Li X (2019). Efficacy of Esketamine Nasal Spray Plus Oral Antidepressant Treatment for Relapse Prevention in Patients With Treatment-Resistant Depression: A Randomized Clinical Trial. *JAMA Psychiatry*.

[34] Nuñez NA, Joseph B, Pahwa M, Kumar R, Resendez MG, Prokop LJ (2022). Augmentation strategies for treatment resistant major depression: A systematic review and network meta-analysis. *Journal of Affective Disorders*.

[35] Phillips JL, Norris S, Talbot J, Hatchard T, Ortiz A, Birmingham M (2020). Single and repeated ketamine infusions for reduction of suicidal ideation in treatment-resistant depression. *Neuropsychopharmacology: Official Publication of the American College of Neuropsychopharmacology*.

[36] McMullen EP, Lee Y, Lipsitz O, Lui LMW, Vinberg M, Ho R (2021). Strategies to Prolong Ketamine’s Efficacy in Adults with Treatment-Resistant Depression. *Advances in Therapy*.

[37] Papakostas GI, Trivedi MH, Shelton RC, Iosifescu DV, Thase ME, Jha MK (2024). Comparative effectiveness research trial for antidepressant incomplete and non-responders with treatment resistant depression (ASCERTAIN-TRD) a randomized clinical trial. *Molecular Psychiatry*.

[38] Rost F, Booker T, Gonsard A, de Felice G, Asseburg L, Malda-Castillo J (2024). The complexity of treatment-resistant depression: A data-driven approach. *Journal of Affective Disorders*.

[39] Ledesma-Corvi S, Jornet-Plaza J, Gálvez-Melero L, García-Fuster MJ (2024). Novel rapid treatment options for adolescent depression. *Pharmacological Research*.

[40] Strawn JR, Aaronson ST, Elmaadawi AZ, Schrodt GR, Holbert RC, Verdoliva S (2020). Treatment-Resistant Depression in Adolescents: Clinical Features and Measurement of Treatment Resistance. *Journal of Child and Adolescent Psychopharmacology*.

[41] Daly EJ, Singh JB, Fedgchin M, Cooper K, Lim P, Shelton RC (2018). Efficacy and Safety of Intranasal Esketamine Adjunctive to Oral Antidepressant Therapy in Treatment-Resistant Depression: A Randomized Clinical Trial. *JAMA Psychiatry*.

[42] Jiang J, Zhang C, Li C, Chen Z, Cao X, Wang H (2021). Magnetic seizure therapy for treatment-resistant depression. *The Cochrane Database of Systematic Reviews*.

[43] Glue P, Loo C, Fam J, Lane HY, Young AH, Surman P (2024). Extended-release ketamine tablets for treatment-resistant depression: a randomized placebo-controlled phase 2 trial. *Nature Medicine*.

[44] Jha MK, Wilkinson ST, Krishnan K, Collins KA, Sanacora G, Murrough J (2024). Ketamine vs Electroconvulsive Therapy for Treatment-Resistant Depression: A Secondary Analysis of a Randomized Clinical Trial. *JAMA Network Open*.

[45] Oliveira-Maia AJ, Bobrowska A, Constant E, Ito T, Kambarov Y, Luedke H (2024). Treatment-Resistant Depression in Real-World Clinical Practice: A Systematic Literature Review of Data from 2012 to 2022. *Advances in Therapy*.

[46] Runia N, Yücel DE, Lok A, de Jong K, Denys DAJP, van Wingen GA (2022). The neurobiology of treatment-resistant depression: A systematic review of neuroimaging studies. *Neuroscience and Biobehavioral Reviews*.

[47] Dunlop K, Talishinsky A, Liston C (2019). Intrinsic Brain Network Biomarkers of Antidepressant Response: a Review. *Current Psychiatry Reports*.

[48] Chabert J, Allauze E, Pereira B, Chassain C, De Chazeron I, Rotgé JY (2022). Glutamatergic and N-Acetylaspartate Metabolites in Bipolar Disorder: A Systematic Review and Meta-Analysis of Proton Magnetic Resonance Spectroscopy Studies. *International Journal of Molecular Sciences*.

[49] Demchenko I, Tassone VK, Kennedy SH, Dunlop K, Bhat V (2022). Intrinsic Connectivity Networks of Glutamate-Mediated Antidepressant Response: A Neuroimaging Review. *Frontiers in Psychiatry*.

[50] Weiss F, Caruso V, De Rosa U, Beatino MF, Barbuti M, Nicoletti F (2023). The role of NMDA receptors in bipolar disorder: A systematic review. *Bipolar Disorders*.

[51] Martinotti G, Dell’Osso B, Di Lorenzo G, Maina G, Bertolino A, Clerici M (2023). Treating bipolar depression with esketamine: Safety and effectiveness data from a naturalistic multicentric study on esketamine in bipolar versus unipolar treatment-resistant depression. *Bipolar Disorders*.

[52] Carhart-Harris RL, Erritzoe D, Williams T, Stone JM, Reed LJ, Colasanti A (2012). Neural correlates of the psychedelic state as determined by fMRI studies with psilocybin. *Proceedings of the National Academy of Sciences of the United States of America*.

[53] Breeksema JJ, Kuin BW, Kamphuis J, van den Brink W, Vermetten E, Schoevers RA (2022). Adverse events in clinical treatments with serotonergic psychedelics and MDMA: A mixed-methods systematic review. *Journal of Psychopharmacology (Oxford, England)*.

[54] Hsieh MH (2023). Electroconvulsive therapy for treatment-resistant depression. *Progress in Brain Research*.

[55] Huang YZ, Edwards MJ, Rounis E, Bhatia KP, Rothwell JC (2005). Theta burst stimulation of the human motor cortex. *Neuron*.

[56] Reddy S, Kabotyanski KE, Hirani S, Liu T, Naqvi Z, Giridharan N (2024). Efficacy of Deep Brain Stimulation for Treatment-Resistant Depression: Systematic Review and Meta-Analysis. *Biological Psychiatry. Cognitive Neuroscience and Neuroimaging*.

[57] Conway CR, Aaronson ST, Sackeim HA, George MS, Zajecka J, Bunker MT (2025). Vagus nerve stimulation in treatment-resistant depression: A one-year, randomized, sham-controlled trial. *Brain Stimulation*.

[58] Scangos KW, Khambhati AN, Daly PM, Makhoul GS, Sugrue LP, Zamanian H (2021). Closed-loop neuromodulation in an individual with treatment-resistant depression. *Nature Medicine*.

[59] Caraci F, Calabrese F, Molteni R, Bartova L, Dold M, Leggio GM (2018). International Union of Basic and Clinical Pharmacology CIV: The Neurobiology of Treatment-resistant Depression: From Antidepressant Classifications to Novel Pharmacological Targets. *Pharmacological Reviews*.

[60] Domke AK, Hempel M, Hartling C, Stippl A, Carstens L, Gruzman R (2023). Functional connectivity changes between amygdala and prefrontal cortex after ECT are associated with improvement in distinct depressive symptoms. *European Archives of Psychiatry and Clinical Neuroscience*.

[61] National Institute for Health and Care Excellence (NICE) (2022). Depression in adults: treatment and management. NICE Guideline NG222. https://www.nice.org.uk/guidance/ng222.

[62] Fabbri C (2025). Treatment-resistant depression: role of genetic factors in the perspective of clinical stratification and treatment personalisation. *Molecular Psychiatry*.

[63] Kang J, Castro VM, Ripperger M, Venkatesh S, Burstein D, Linnér RK (2024). Genome-Wide Association Study of Treatment-Resistant Depression: Shared Biology With Metabolic Traits. *The American Journal of Psychiatry*.

[64] Del Casale A, Pomes LM, Bonanni L, Fiaschè F, Zocchi C, Padovano A (2022). Pharmacogenomics-Guided Pharmacotherapy in Patients with Major Depressive Disorder or Bipolar Disorder Affected by Treatment-Resistant Depressive Episodes: A Long-Term Follow-Up Study. *Journal of Personalized Medicine*.

[65] Cheng Y, Liu H, Yuan R, Yuan K, Yu S (2023). Effectiveness of pharmacogenomics on the response and remission of treatment-resistant depression: a meta-analysis of randomised controlled trials. *General Psychiatry*.

[66] Fonseka TM, MacQueen GM, Kennedy SH (2018). Neuroimaging biomarkers as predictors of treatment outcome in Major Depressive Disorder. *Journal of Affective Disorders*.

[67] Gonda X, Dome P, Neill JC, Tarazi FI (2023). Novel antidepressant drugs: Beyond monoamine targets. *CNS Spectrums*.

[68] Pettorruso M, Di Lorenzo G, Benatti B, d’Andrea G, Cavallotto C, Carullo R (2024). Overcoming treatment-resistant depression with machine-learning based tools: a study protocol combining EEG and clinical data to personalize glutamatergic and brain stimulation interventions (SelecTool Project). *Frontiers in Psychiatry*.

