# Supplementary material for: Rethinking Treatment-Resistant Depression: A Systematic Review of Novel Therapeutic Strategies and Precision Medicine Approaches
Source: Actas Esp Psiquiatr. 2025 Dec 17;53(6):1395–409. doi: 10.62641/aep.v53i6.1946 (PMC12728552; doi:10.62641/aep.v53i6.1946)
Supplement: Supplementary file 1 [file ActEsp-53-6-1395-1409-s1.zip › Supplementary Table 1.docx]

**Supplementary Table 1:** Search Terms and Boolean Operators

| Database | Search Terms Used | Boolean Operators | Filters Applied |
| --- | --- | --- | --- |
| PubMed | ("treatment-resistant depression" OR " bipolar disorder" OR “depressive episode” OR “mood disorder spectrum”) AND (“treatment response” OR “antidepressant resistance” OR “augmentation therapy” OR “mood stabilizers” OR “atypical antipsychotics”) | AND/OR | Peer-reviewed articles, English, Humans, Full-text available, 2015-2025, Preprints Excluded |
| Scopus | ("treatment-resistant depression" OR " bipolar disorder" OR “depressive episode” OR “mood disorder spectrum”) AND (“treatment response” OR “antidepressant resistance” OR “augmentation therapy” OR “mood stabilizers” OR “atypical antipsychotics”) | AND/OR | Peer-reviewed articles, English, Humans, Full-text available, 2015-2025, Preprints Excluded |
