# Supplementary material for: Rethinking Treatment-Resistant Depression: A Systematic Review of Novel Therapeutic Strategies and Precision Medicine Approaches
Source: Actas Esp Psiquiatr. 2025 Dec 17;53(6):1395–409. doi: 10.62641/aep.v53i6.1946 (PMC12728552; doi:10.62641/aep.v53i6.1946)
Supplement: Supplementary file 1 [file ActEsp-53-6-1395-1409-s1.zip › Supplementary Table 2.docx]

**Supplementary Table 2.** Pharmacological, Psychotherapeutic, and Neuromodulation Treatments Used in Studies Included in the Review

| Study | Drug | Manufacturer | Location | Batch Number |
| --- | --- | --- | --- | --- |
| Zengin et al. (2022) [25] | rTMS | Not specified | Not specified | Not specified |
| Scott et al. (2023) [26] | Various pharmacological treatments (e.g., antidepressants, antipsychotics, mood stabilisers) | Multiple manufacturers | Various locations | Not specified​ |
|  | Various psychotherapeutic treatments (e.g., CBT, DBT, IPT) | Not applicable | Not applicable | Not applicable |
| Palhano-fontes et al. (2019) [27] | Ayahuasca | Traditional Preparation | Brazil | Not specified |
| Zakhour et al. (2020) [28] | CBT | Not applicable | Not applicable | Not applicable |
| Fedgchin et al. (2019) [29] | Esketamine Nasal Spray | Spravato®, Janssen Pharmaceuticals | Titusville, NJ, USA | Not specified |
| Cladder-micus et al. (2018) [30] | MBCT | Not applicable | Not applicable | Not applicable |
| Ijaz et al. (2018) [31] | Psychological Therapies (CBT, IPT, DBT) | Not applicable | Not applicable | Not applicable |
| Lenze et al. (2023) [32] | Aripiprazole | Abilify^®^, Otsuka Pharmaceuticals | Tokyo, Japan | Not specified |
|  | Bupropion (Extended-Release) | Wellbutrin XL^®^, GlaxoSmithKline | Brentford, UK | Not specified |
|  | Lithium | Not specified | Not specified | Not specified |
|  | Nortriptyline | \| Pamelor™, Mallinckrodt Pharmaceuticals \| \| --- \| | \| Staines-upon-Thames, UK \| \| --- \| | Not specified |
| Daly et al. (2019) [33] | Esketamine Nasal Spray | Spravato®, Janssen Pharmaceuticals | Titusville, NJ, USA | Not specified |
| Nuñez et al. (2022) [34] | Various augmentation strategies | Not specified | Not specified | Not specified​ |
| Phillips et al. (2020) [35] | Ketamine (IV) | Hikma Pharmaceuticals | London, UK | Not specified​ |
| Mcmullen et al. (2021) [36] | Ketamine (IV) | Hikma Pharmaceuticals | London, UK | Not specified​ |
| Papakostas et al. (2024) [37] | Aripiprazole | Abilify^®^, Otsuka Pharmaceuticals | Tokyo, Japan | Not specified​ |
|  | Venlafaxine XR | Effexor XR®, Wyeth | Collegeville, PA, USA | Not specified​ |
|  | rTMS | Not specified​ | Not specified​ | Not specified​ |
| Rost et al. (2024) [38] | Observational study | Not applicable | Not applicable | Not applicable​ |
| Ledesma-corvi et al. (2024) [39] | Various pharmacological treatments (e.g., ketamine, esketamine, psychedelics, cannabinoids) | Not specified | Not specified | Not specified​ |
|  | Various neuromodulation treatments (e.g., rTMS, ECT, DBS) | Not specified​ | Not specified​ | Not specified​ |
| Strawn et al. (2020) [40] | Observational study | Not applicable | Not applicable | Not applicable​ |
| Daly et al. (2018) [41] | Esketamine Nasal Spray | Spravato®, Janssen Pharmaceuticals | Titusville, NJ, USA | Not specified |
| Jiang et al. (2021) [42] | MST | Mecta Corp. | Tualatin, OR, USA | Not specified |
| Glue et al. (2024) [43] | Extended-Release Ketamine | ATAI Life Sciences | Berlin, Germany | Not specified |
| Jha et al. (2024) [44] | Ketamine (IV) | Hikma Pharmaceuticals | London, UK | Not specified |
| Oliveria-maia et al. (2024) [45] | Real-world outcomes study; Various pharmacological, psychotherapeutic and neuromodulation treatments | Not specified | Not specified | Not specified |

rTMS: repetative Transcranial Magnetic Stimulation; CBT: Cognitive Behavioral Therapy; DBT: Dialectical Behavior Therapy; IPT: Interpersonal Psychotherapy; MBCT: Mindfulness-Based Cognitive Therapy; ECT: Electroconvulsive Therapy; DBS: Deep Brain Stimulation; IV: Intravenous
