# Supplementary material for: Rethinking Treatment-Resistant Depression: A Systematic Review of Novel Therapeutic Strategies and Precision Medicine Approaches
Source: Actas Esp Psiquiatr. 2025 Dec 17;53(6):1395–409. doi: 10.62641/aep.v53i6.1946 (PMC12728552; doi:10.62641/aep.v53i6.1946)
Supplement: Supplementary file 1 [file ActEsp-53-6-1395-1409-s1.zip › Supplementary Table 3.docx]

Supplementary Table 3. Risk of Bias Assessment for the 21 Included Studies

| Study | Study Type | RoB Tool | Overall Risk Of Bias | Comments |
| --- | --- | --- | --- | --- |
| Zengin et al. (2022) [25] | RCT | Cochrane RoB 2 | High | Crossover design without washout-potential carryover/period effects. Baseline imbalance in lithium use; small sample; ITT not clearly reported; blinding applied but sensory unblinding is possible. |
| Scott et al. (2023) [26] | Systematic Review & Meta-Analysis | ROBIS | Some concerns | Restricted database coverage; limited grey literature search; protocol deviations; high heterogeneity; publication bias not fully assessed. |
| Palhano-fontes et al. (2019) [27] | RCT | Cochrane RoB 2 | Some concerns | Randomization and blinding reported; trial registered. Small sample; the strong psychoactive profile may compromise blinding; concomitant benzodiazepine use is a potential confounder. |
| Zakhour et al. (2020) [28] | Systematic review | RCT-PQRS | NI | PRISMA-guided systematic review of seven databases including RCTs, open-label trials, and one case report. Risk of bias was evaluated only for the RCTs using RCT-PQRS (all rated low); no review-level ROBIS/AMSTAR assessment was reported. |
| Fedgchin et al. (2019) [29] | RCT (Phase 3) | NR | Low | Computer-generated randomization with stratification; identical nasal spray devices; placebo with bittering agent; independent blinded remote raters; modified ITT (≥1 dose). |
| Cladder-Micus et al. (2018) [30] | RCT | NR | Some concerns | Open-label design; primary outcome self-reported; remission interviews not blinded to allocation (with blinded reliability check on a subsample); relatively high non-completion in MBCT arm; ITT reported. |
| Ijaz et al. (2018) [31] | Systematic review | Cochrane RoB 2 (for included RCTs); review-level ROBIS: NR | NI | Allocation concealment unclear in one trial; low risk in the other five trials. Detection bias high for self-reported outcomes but largely low for observer-rated outcomes. GRADE mostly moderate for short-term symptoms; dropout rates similar between groups. |
| Lenze et al. (2023) [32] | Open-label RCT | Cochrane RoB 2 | Some concerns | Open-label; patients/investigators aware, outcome assessors blinded; ITT reported. |
| Daly et al. (2019) [33] | RCT (Phase 3) | Cochrane RoB 2 | Low | Double-blind with matched bittered placebo device; independent blinded remote raters. |
| Nuñez et al. (2022) [34] | Systematic Review & Network Meta-Analysis | Cochrane RoB 2 (for included RCTs); review-level ROBIS: NR | NI | PRISMA; multi-database search; study-level RoB mostly moderate (some high, few low); publication bias not detected; review-level ROBIS not reported. |
| Phillips et al. (2020) [35] | RCT + open-label (secondary analysis) | NR | Some concerns | Phase 1: crossover design; outcome raters blinded; benzodiazepines held on infusion days.  Phases 2–3 open-label; small sample; possible sensory unblinding due to psychoactive effects. |
| Mcmullen et al. (2021) [36] | Systematic review | Review-level ROBIS/AMSTAR: NR | NI | PRISMA; single-database search; heterogeneity across designs/doses/frequencies noted; review-level RoB not reported. |
| Papakostas et al. (2024) [37] | RCT | Cochrane RoB 2 | Some concerns | Computer-generated randomization; blinded raters; open-label to patients/investigators; efficacy analyses on modified ITT. |
| Rost et al. (2024) [38] | Observational descriptive analysis | NR | NI | Observational cross-sectional baseline report with no formal RoB appraisal. Unblinded, descriptive analyses; recall bias and no control group acknowledged. |
| Ledesma-corvi et al. (2024) [39] | Narrative review | NR | NI | Narrative review; ROBIS/AMSTAR not reported. |
| Strawn et al. (2020) [40] | Observational cross-sectional baseline analysis | NR | NI | Observational, cross-sectional baseline report with no formal RoB assessment. Confounding/selection not controlled; no control group; recall/self-report bias acknowledged; assessments unblinded. |
| Daly et al. (2018) [41] | RCT (Phase 2) | Cochrane RoB 2 | Some concerns | Computer-generated randomization; double-blind with bittered placebo device; ITT stated; short 1‑week periods and small sample; potential sensory unblinding due to psychoactive effects. |
| Jiang et al. (2021) [42] | Systematic Review | Cochrane RoB 2 for included RCTs; review-level ROBIS: NR | NI | Across included RCTs: no blinding, unclear allocation concealment in at least one trial, non-validated outcome measures, baseline imbalances/skewed data, and device-manufacturer funding noted. Review-level ROBIS not reported. |
| Glue et al. (2024) [43] | RCT (Phase 2) | NR | Some concerns | Randomized-withdrawal after open-label enrichment (responders only); double-blind with home dosing; high discontinuation (risk of missing outcome data); psychoactive effects may challenge blinding. |
| Jha et al. (2024) [44] | RCT | NR | Some concerns | Open-label; self-reported primary outcome; secondary non-prespecified analysis; modified ITT (≥1 post-baseline assessment); centralized web randomization (adequate concealment). |
| Oliveria-Maia et al. (2024) [45] | Systematic Review | AHFMR quality tool for included observational studies; review-level ROBIS/AMSTAR: NR | NI | Included observational studies were appraised with the AHFMR quality tool; no unified review-level RoB judgement provided. |

**Risk-of-bias (RoB) assessment.** For RCTs, we used Cochrane RoB 2 (individual/cluster/crossover variants). For systematic reviews, we used ROBIS (review-level). For observational studies, we used ROBINS-I (or, when explicitly reported by the authors, the stated quality tool, e.g., AHFMR). For psychotherapy RCTs, when authors reported RCT-PQRS, this is noted in the table. **Judgements.** *Low*, *Some concerns*, *High*. NI: no information; NR: not reported. Unless otherwise specified, RoB 2 judgements are summarized for the study’s primary outcome.

**Abbreviations:** RCT: Randomized Controlled Trial; PRISMA: Preferred Reporting Items for Systematic Reviews and Meta-Analyses; RoB: Risk of Bias; ROBIS: Risk Of Bias In Systematic reviews; ROBINS-I: Risk Of Bias In Non-randomized Studies of Interventions; RCT-PQRS: Randomized Controlled Trial – Psychotherapy Quality Rating Scale; AHFMR: Alberta Heritage Foundation for Medical Research quality tool.
